# Effect of JumpstartMD, a Commercial Low-Calorie Low-Carbohydrate Physician-Supervised Weight Loss Program, on 22,407 Adults

**DOI:** 10.1155/2020/8026016

**Published:** 2020-01-21

**Authors:** Sean Bourke, John Magaña Morton, Paul Williams

**Affiliations:** ^1^JumpstartMD, 350 Lorton Ave, Burlingame, CA 94010, USA; ^2^Department of Surgery, Stanford University Medical Center, Stanford, CA, USA; ^3^Childrens Hospital Oakland Research Institute, Oakland, CA, USA

## Abstract

**Background:**

Commercial weight loss programs provide valuable consumer options for those desiring support. Several commercial programs are reported to produce ≥3-fold greater weight loss than self-directed dieting. The effectiveness of JumpstartMD, a commercial pay-as-you-go program that emphasizes a low-to-very-low-carbohydrate real-food diet and optional pharmacologic treatment without prepackaged meals or meal replacement, has not previously been described.

**Methods:**

Completer and last observation carried forward (LOCF) of clinic-measured weight loss (kg) in 18,769 female and 3638 male JumpstartMD participants.

**Results:**

Completers lost (mean ± SE) 8.7 ± 0.04 kg, 9.5 ± 0.04% with 44.5 ± 0.5% achieving ≥10% weight loss at 3 months (mo, *N* = 14,999 completers); 11.8 ± 0.1 kg, 12.6 ± 0.1% with 66.4 ± 0.6% achieving ≥10% weight loss at 6 mo (*N* = 11,805); and 11.5 ± 0.2 kg, 12.0 ± 0.2% with 57.6 ± 0.9% achieving ≥10% weight loss at 12 mo (*N* = 8514). LOCF estimates were −6.5 ± 0.03 kg, −7.2 ± 0.03% with 27.1 ± 0.3% achieving ≥10% weight loss at 3 mo; −7.7 ± 0.04 kg, −8.5 ± 0.04% with 36.3 ± 0.3% achieving ≥10% weight loss at 6 mo; and −7.7 ± 0.1 kg, −8.4 ± 0.1% with 34.6 ± 0.3% achieving ≥10% weight loss after 12 mo. Frequent health coach meetings was a major determinant of weight loss, with women and men attending ≥75% of their weekly appointments losing 8.8 ± 0.04 and 11.9 ± 0.1 kg, respectively, after 3 mo, 13.1 ± 0.1 and 16.5 ± 0.3 kg after 6 mo, and 16.5 ± 0.3 and 19.4 ± 0.8 kg after 12 mo. Phentermine and phendimetrazine had a minor effect in women only at 1 (6.1% greater weight loss than untreated), 2 (4.1%), and 3 mo (1.2%), but treated patients showed longer enrollment than nontreated during the first 3 (females: +0.4 ± 0.01; males: +0.3 ± 0.04 mo), 6 (females: +1.1 ± 0.04; males: +1.0 ± 0.1 mo), and 12 mo (females: +2.7 ± 0.1; males: +2.4 ± 0.2 mo). JumpstartMD produced generally greater weight loss than published reports for other real-food and prepackaged-meal commercial programs and somewhat greater or comparable losses to meal replacement diets.

**Conclusion:**

A one-on-one medically supervised program that emphasized real low-carbohydrate foods produced effective weight loss, particularly in those attending ≥75% of their weekly appointments.

## 1. Introduction

Seventy percent of US adults are overweight or obese [1], 42% report trying to lose weight, and 23% report trying to maintain weight annually [2]. Five to ten percent weight loss produces clinically significant health benefits [3], with even greater health benefits likely to accrue with greater weight loss [4].

Americans spend 2.5 billion dollars annually on commercial weight loss programs, broadly categorized as calorie-counting, food choice, and meal plans (e.g., Weight Watchers, Slimming World, Rosemary Conley, Biggest Losers Club, Metabolic Balance Nutrition Program and Itrim), prepackaged meals (e.g., Jenny Craig and Diet Chef), and meal replacement products including liquid shake diets (e.g., Nutrisystem, Medifast, Health Management Resource, and Itrim). Weight Watchers represent 45% of market, Nutrisystem 14%, and Jenny Craig 13%, with a number of other programs making up the remaining share [5]. Slimming World and Rosemary Conley are available as online programs in the United States. In Europe, Australia, and some US states, partnerships exist between national payers and these programs for patients seeking weight loss. Several commercial programs have been shown to produce twice as much weight loss as those administered through standard healthcare settings [6, 7], and ≥3-fold greater weight loss than self-directed dieting [8, 9]. The odds for >10% weight loss in obese NHANES participants trying to lose weight were 70% greater for those that joined a commercial weight loss program vs. self-directed weight loss [10].

JumpstartMD's approach to weight management uses a real-food low-to-very-low-carbohydrate diet and one-on-one, personalized instruction and support from trained health counselors in the setting of a medical clinic. It is an alternative to other commercial calorie-counting and meal plan programs that use group education and group support. This report presents weight loss data from over 20,000 self-paying adults who participated in the JumpstartMD program in the San Francisco Bay Area between 2007 and 2017. Uncontrolled longitudinal patient data have previously been reported in peer-reviewed journals for Weight Watchers [11–14], Slimming World [12, 13, 15–22], Rosemary Conley [12, 13], Jenny Craig [23, 24], Biggest Loser Club [25], Itrim [26], Metabolic Balance Nutrition Program [27], Medifast [28, 29], and Health Management Resources [30]. Mean weight loss for JumpstartMD patients are compared to the published weight losses of other commercial programs, taking into account covariates reported to affect weight loss success: BMI, sex, and age.

## 2. Methods

JumpstartMD is a pay-as-you-go weight management program supervised by physicians that emphasizes the benefits of a low-carbohydrate diet and healthy lifestyle. Through weekly one-on-one clinic visits personalized to fit each person's life and by emphasizing fresh, whole, nonpackaged foods, JumpstartMD's health coaches provided accountability and support, nutritional guidance, behavioral modification, and fitness recommendations to achieve sustainable wellness goals. Because the program is medically supervised and a licensed healthcare practitioner is present in every center, JumpstartMD is also capable of making adjustments in diabetes, blood pressure, and other medications as well as offering FDA-approved weight loss medications for interested and eligible patients. Participants attended the program for as long as they wished.

Weight loss was achieved through nutritional guidance, tracking, and meal planning to create caloric deficits for sustained weight loss without the use of prepackaged foods or meal replacements. Clinicians prescribed a recommended nutritional guide path for members early in their program that eliminated consumption of grains, starches, and sugars while limiting consumption of carbohydrates to typically under 50 grams a day from low glycemic (nonstarchy) vegetables and fruits. Dietary carbohydrate recommendations varied by participant based on starting weight and degree of insulin resistance as noted from baseline laboratory tests. Structured meal plans were prescribed using portion sizes. Following the recommendations provided, participants' energy intake derived from carbohydrate would typically fall between 10% and 20%, and sometimes as low as 5%. While JumpstartMD does not specifically “count calories” or discuss “calories consumed,” properly followed, the program would typically generate a dietary caloric deficit on the order of 500 to 1000 Calories or more per day.

Dietary compliance was tracked using the program's pocket-sized forms or the participant's own mobile phone-based software, which were reviewed weekly with their health coach or clinician. Dietary carbohydrate intake was monitored by measuring and recording amounts of approved fruits and vegetables consumed. Dietary protein consumption was similarly monitored by measuring and recording consumption of foods with high protein content, including meats and seafoods, dairy products, and legumes. Healthy dietary fats were tracked and monitored only when the food's primary macronutrient composition was fat (i.e., as in the case of butter, cream, olive oil, nuts, seeds, and avocado). Dietary tracking was an important component of the intervention but was not collected in a standardized manner for data analysis.

Physical activity recommendations generally correspond to current Centers for Disease Control (CDC) recommendations while also supporting the patient's own physical activity goals. Weights were measured directly at each clinic visit in light clothing without shoes (Tanita digital healthcare scales; Arlington Heights, IL). Participants were provided a macronutrient tracking tool to help them record their daily consumption of allotted carbohydrate, protein, and healthy fat servings. Participants reviewed their progress in achieving their macronutrient recommendations with their individual JumpstartMD clinician or health coach via weekly one-on-one in person visits. The program has expanded from a single clinic in 2007 to thirteen clinics across the greater San Francisco Bay Area.

### 2.1. Statistical Analysis

Anonymized data were provided to an independent statistician not previously associated with the commercial program for analysis. Attendance was calculated at each visit as the ratio of the number of follow-up appointments attended divided by the expected number of weekly appointments (i.e., number of days since baseline starting date divided by seven). For each individual, average number of days since baseline, weight loss, % weight loss, BMI change, attendance, and ≥5% and ≥10% weight loss were calculated within each two-week interval since baseline. Mean changes from baseline ± SE were calculated over all subjects within each interval and plotted vs. the average follow-up duration. Regression analyses were used to assess the relationship between weight loss vs. attendance, baseline BMI, sex, and age. Analysis of covariance was used to assess weight loss differences between patients on and off obesity drugs, with and without adjustment for enrollment duration. Results are presented with standard deviations given in parentheses “(SD)” or ±standard errors (±SE). Standard errors were reported because they represent the precision of the estimates (standard deviations can be computed by multiplying the SE by the square root of the sample size). Last observation carried forward (LOCF) included all subjects with follow-up weights and was calculated at 3, 6, 9, and 12 months. Interpolation was used to estimate results for completers at 1 month (30.4 days), 3 months (91.25 days), 6 months (182.5 days), 9 months (273.75 days), and one year (365 days). High-attendance completers were defined as patients attending at least 75% of their weekly appointments, as has been defined by others [14, 22, 30]. All analyses were performed using JMP version 13.2.0 (SAS institute, Cary, NC). Race, socioeconomic status, and comorbidity data were not collected.

### 2.2. Comparison with Other Published Studies

JumpstartMD weight losses were compared to those reported for other commercial programs (Weight Watchers [6, 8, 11–14, 31–42], Slimming World [12, 13, 15–22, 37, 43], Rosemary Conley [12, 13, 37, 42], Biggest Losers Club [25, 44, 45], eDiet [46, 47], Jenny Craig [23, 24, 33, 48–50], Itrim [26], Nutrisystem [9, 33, 51–53], Medifast [28, 29, 54–56], Health Management Resources [30, 57–59], Metabolic Balance Nutrition Program [27], and Diet Chef [60]) when matched for follow-up duration, BMI eligibility, age, proportion of males, and sex-specific baseline BMI effects. The studies were identified from systematic reviews, PubMed searches by program name, and references cited within each paper retrieved (Table 1). We excluded studies that used commercial weight loss products but were directed by others (e.g., Slimfast and Optifast) because they lacked standardized behavioral interventions. We also excluded very-low-calorie diets, studies conducted before the year 2000, studies not reporting LOCF or completers, and studies in cancer survivors.

Results are presented in as forest plots with diamonds representing the average differences between JumpstartMD and other commercial programs (identical size for each comparison) and 95% confidence intervals for the differences. Standard errors (SE) were taken from the published reports, calculated as SD/N^0.5^, or calculated from the 95 percent confidence intervals as (upper-lower bound)/(2*∗*1.96). When SEs were not otherwise available, the standard deviations were estimated from the relationship between mean weight change (independent variable) and the standard deviation of weight change (dependent variable) from the JumpstartMD dataset, using the coefficients of Table 2. Different coefficients were used for estimating “LOCF” and “completer” standard deviations. Median weight losses [11, 14, 32] were increased by 5.2% for weight change (∆kg), 2.1% for percent weight change (∆%), and 4.4% for BMI change (∆kg/m^2^) corresponding to the JumpstartMD difference between mean weight loss and median weight losses.

### 2.3. Statistical Methods, LOCF

The primary outcome for comparing JumpstartMD to other commercial programs was LOCF because it encapsulates both retention and weight loss success. Differences in design and analyses have stymied prior efforts to synthesize commercial weight loss results by meta-analyses [61]. Our analyses adjust for these differences by tailoring the JumpstartMD sample to the recruitment specifications and follow-up duration of each published report. Specifically, adjusted JumpstartMD LOCF weight losses (mean ± SE_prediction_) were calculated to corresponding to the average baseline BMI, age, and proportion of men of each commercial program. This was done by (1) restricting the JumpstartMD data to the commercial study's BMI recruitment range and (2) applying multiple linear regression to the restricted JumpstartMD LOCF data with age, male sex, and sex-specific baseline BMI effects as independent variables. Prediction formulas for the expected value and the prediction standard errors were then evaluated using the average age, sex, and BMI for the reported study (Table 1). The mean differences between JumpstartMD and the reported commercial studies and the standard error for the difference were estimated by standard methods.

The JumpstartMD LOCF analysis included only those subjects who chose to continue their program beyond their initial assessment visit. Some published studies assigned zero weight loss to patients without any follow-up data, affecting the denominator but not the numerator of the average LOCF. Ahern et al. reported that the proportion of the sample lacking follow-up data was 5.35% (1570/29326) [11], Aston et al. reported 7.0% (74/1058) [32], Heshka et al. 6.1% (13/211) [8], Stubbs et al. 6.8% out of 1.43 million [22], Martin et al. approximately 6% out of 81,505 [24], Furlow and Anderson reported 5.9% (12/204) [30], Hutchesson et al. reported 2.2% for standard and 2.4% for fast-track challenge [25], and 2.2% standard rate assumed to apply to Collins et al. [45]. Avery et al. reported 997 of 1020 Healthy Living Pharmacy patients and 5306 of 5482 general practitioner patients returned for follow-up weigh-ins [16]. Therefore, to achieve comparability to the JumpstartMD results, the reported estimates from other studies were adjusted upwards by decreasing their denominator by the proportion of the sample that lacked follow-up (assumed to be 7% unless otherwise stated [6, 12, 20, 37]). An anomaly was Mitchell et al. who reported 25.7% non-follow-up among 1605 Medicaid recipients, which may be due to the lower socioeconomic status or racial composition of their sample [14]. To obtain corresponding samples, JumpstartMD LOCF data were calculated for patients attending ≥4 weeks for comparison with Meffert and Gerdes [27].

The recruitment criteria for subsidized physician referrals were generally ≥30 kg/m^2^, but exceptions could be made per the physician's discretion [20]. Therefore, for comparisons with subsidized physician referrals, the JumpstartMD sample included randomly selected overweight patients (25 < BMI < 30) to match their reported recruitment proportion (assumed to be 4% as reported by Dixon et al. [12] unless otherwise specified).

Follow-up duration was taken as that described by the commercial study, i.e., 84 days if described as 12-week weight loss, 91.25 days if described as 3-month weight loss, or if otherwise described (e.g., 12 meetings or until a 5-week hiatus [11]). Although the 12 weekly Weight Watcher or Slimming World meetings represent only 11-week differences, patients apparently had 14 weeks to complete the course [20, 21] and therefore were compared to 12-week JumpstartMD weight changes.

### 2.4. Statistical Methods, Completers

JumpstartMD completer weight losses were compared to those reported for other commercial studies when matched for follow-up duration, recruitment BMI range, baseline age, proportion of males, and sex-specific baseline BMI effects as described in Methods section. Specifically, we (1) selected the JumpstartMD subset corresponding to the BMI range of the other commercial program, (2) divided the JumpstartMD records into 2-week intervals, (3) obtained the regression coefficients for expected weight loss and SE_prediction_ within each interval by least-squares regression with age, sex, and sex-specific effects of baseline BMI as independent variables, (4) within each interval, used these regression coefficients to calculate the expected weight loss and SE_prediction_ corresponding to the average baseline age, sex, and BMI of the other commercial program, and (5) used linear interpolation to estimate weight loss for the designated length of follow-up from the proximal 2-week intervals. Comparisons are presented as “(JumpstartMD ± SE_prediction_ vs. other commercial program ± SE).” In some cases [13, 43, 44, 50, 60], weight loss for completers was calculated from baseline observation carried forward (BOCF) by inflating the BOCF weight loss by the ratio of the total sample divided by the number of patients providing data (i.e., zero weight loss in patients lost to follow-up increases the denominator but not the numerator in calculating mean weight loss).

Completers were usually defined as participants with a weight measurement at a specified follow-up duration; however, Stubbs et al. defined “completers” as completing 10^th^, 11^th^, or 12^th^ visit by the 14^th^ week [20] and the 20^th^, 21^st^, 22^nd^, 23^rd^, or 24^th^ visit by the 28^th^ week [21]. Aherns et al. defined completers as completing 12 visits or the last observation until a 5-week hiatus in attendance [11]. JumpstartMD completers were correspondingly defined for comparisons with these studies. Additional adjustments for the inclusion of non‐obese patients and the estimation of 12-week follow-up for Weight Watchers and Slimming World were as described above for LOCF.

### 2.5. Meta-Analysis

Pooled estimates were calculated as weighted averages of the inverse of the standard error^2^ (fixed effect meta-analysis).

## 3. Results

Of the 24,395 patients in the electronic patient base, we excluded 114 for errors in the admission date (prior to Jan 1, 2007) or missing sex information and 59 for missing baseline body weights. Of the 24,222 patients remaining, we excluded 1815 (7.5%) patients who received an initial evaluation and weight loss recommendations but chose not to enroll with the program, leaving 22,407 patients for the analysis of weight change.

Women represented 83.8% of the participating patient population. On average, they were slightly younger than the men (mean (SD): 51.8 (12.0) vs. 53.7 (12.4) years, *P* < 10^−16^), including a higher percent (±SE) under 40 years (women vs. men: 15.6 ± 0.3 vs. 12.9 ± 0.6%), and between 40 and 64 years (70.2 ± 0.3 vs. 67.4 ± 0.8%), and a lower proportion 65 years and older (14.1 ± 0.3 vs. 19.7 ± 0.7%). Women also had lower BMI than men (31.2 (5.8) vs. 34.3 (5.3) kg/m^2^, *P* < 10^−16^). For the sexes combined, 10.1% were healthy weight, 33.9% overweight, 31.1% Class I (30 ≤ BMI < 35 kg/m^2^), 15.2% Class II (35 ≤ BMI < 40 kg/m^2^), and 9.5% Class III obese (BMI ≥ 40 kg/m^2^).

### 3.1. Retention

**Figure 1:**
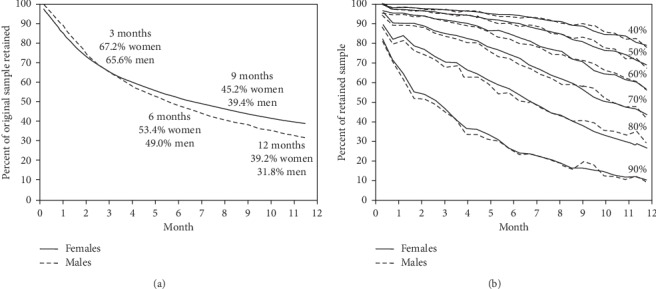
(a) Retention (as a percentage of all 22,407 participants who enrolled) and (b) attendance (as a percentage of completers at each time point). JumpstartMD patients had a financial enticement to terminate the program early, which is not the case for clinical trials or subscription weight loss programs. Specifically, clinical trials subjects consent to participate in a no-cost program for a specified duration and subscribers prepay for a specified program length. In contrast, JumpstartMD patients pay a significant fee to receive their initial evaluation and recommendations and separately pay significant fees to participate on a month-to-month basis without obligation for as long as they perceive benefit from the program.

Figure 1(a) shows that approximately two-third of the 18,769 women (67.2%) and 3638 men (65.6%) attended ≥3 months, 53.4% of the women and 49% of the men attended ≥6 months, 45.2% of the women and 39.4% of the men attended ≥9 months, and 39.2% of the women and 31.8% of men attended for ≥one year. Regression analyses showed that during the first 3, 6, 9, and 12 months, women were enrolled an average ± SE of 1.2 ± 0.5, 4.9 ± 1.2, 10.3 ± 2.0, and 16.4 ± 2.7 days longer than men, respectively, and each year of age was associated with 0.17 ± 0.01, 0.47 ± 0.04, 0.81 ± 0.06, and 1.12 ± 0.08 days additional enrollment, respectively. The effects of BMI on enrollment duration were not significant at 9 and 12 months.

### 3.2. Attendance

Figure 1(b) displays the intensity of participation as measured by clinic attendance. Among completers, the percent of patients attending at least 50%, 60%, 75%, and 90% of clinic appointments was 95.1%, 90.8%, 78.0%, and 45.9% during the first three months, respectively; 89.5%, 82.7%, 62.6%, and 24.6% during the first six months, respectively; 80.3%, 68.9%, 45.1%, and 27.6% during the first nine months, respectively; and 66.5%, 53.3%, 30.8%, 8.3% for the year (percentages are the cumulative average attendance through the patient's last visit). Regression analyses showed that during the first 3, 6, 9, and 12 months, percent attendance averaged ± SE 2.10 ± 0.43%, 4.0 ± 0.65%, 3.49 ± 0.93%, and 5.40 ± 1.17% higher in women than men, respectively, and averaged 0.30 ± 0.03%, 0.76 ± 0.0.04%, 1.09 ± 0.06%, and 1.34 ± 0.07% higher per kg/m^2^ increment in BMI, respectively. Age was not significantly related to attendance.

### 3.3. Weight Loss in Completers

**Figure 2:**
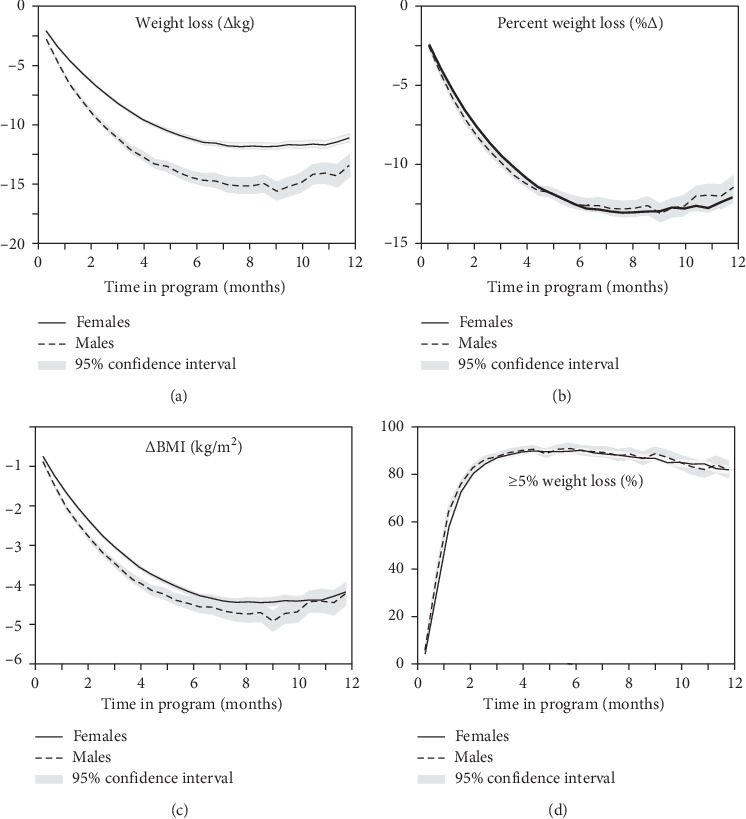
Changes in weight (∆kg), percent change in weight, BMI (∆kg/m^2^), and percent of patients with ≥5% weight loss in JumpstartMD patients over time. Shaded areas designate 95% confidence interval. Sample sizes were 18,769 women and 3686 for all variables except BMI, which were 18115 women and 3528 men due to missing heights.

**Figure 3:**
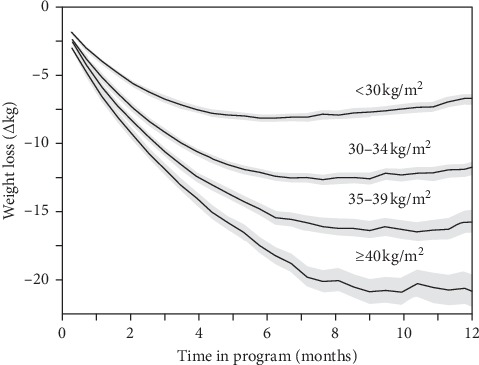
Change in weight (∆kg) over time by baseline BMI. There were 9525 healthy or overweight patients (<30 kg/m^2^), 6776 class I obese patients (30–34 kg/m^2^), 3295 class II obese patients (35–39 kg/m^2^), and 2043 class III obese patients (≥40 kg/m^2^) at baseline.

Figure 2 presents the average weight loss in completers regardless of their level of active participation or starting weight. At the end of the first month, the patients lost an average of 4.37 ± 0.02 kg and 4.86 ± 0.02% of body weight and 1.57 ± 0.01 kg/m^2^ of BMI. Nearly one-half (46.59 ± 0.33%) had lost over 5% of their body weight. At the end of 13 weeks, the patients lost an average of 8.66 ± 0.04 kg body weight, representing a 9.47 ± 0.04% reduction, and a 3.12 ± 0.01 kg/m^2^ drop in BMI, with 86.3 ± 0.31% attaining ≥5% and 44.51 ± 0.45% attaining ≥10% weight loss. After 6 months (26 weeks), the completers had lost 11.81 ± 0.08 kg, 12.57 ± 0.08% of baseline weight, and 4.28 ± 0.03 kg/m^2^. Ninety percent (89.73 ± 0.37%) had lost over 5% and 66.37 ± 0.58% had lost over 10% of body weight. Thereafter, weight loss leveled off, with those completing 9 months (39 weeks) averaging 12.39 ± 0.13 kg weight loss, 12.96 ± 0.12% weight loss, and 4.52 ± 0.05 kg/m^2^ reduction in their BMI from baseline. Five percent or greater weight loss was attained by 87.13 ± 0.52% of the sample and 10% or greater weight loss by 65.32 ± 0.74% of completers. Similarly, at one year, average weight loss was 11.45 ± 0.16 kg, 11.98 ± 0.16%, and 4.22 ± 0.06 kg/m^2^ with 81.37 ± 0.70% losing ≥5% body weight and 57.57 ± 0.89% weight loss losing ≥10% body weight.  Figure 3 shows that the amount of weight loss was strongly related to baseline BMI.

**Figure 4:**
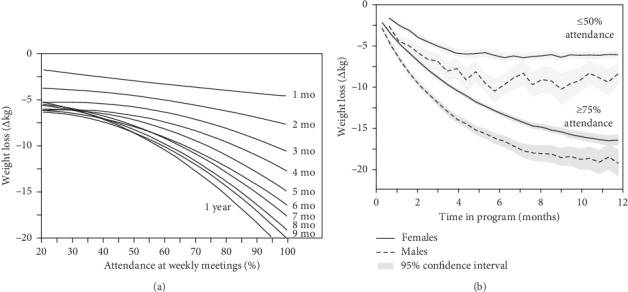
Effects of patient participation on weight loss. (a) The effect of average cumulative attendance on weight change at month 4, month 5,…, 1 year in men and women combined. (b) The average weight loss by month in the high attendees (≥75 attendance at weekly appointments) vs. patients who attended <50% of weekly healthcare coach appointments (low attendance) in men and women separately.

Figure 4 shows that weight loss success depended on regular meetings with the patients' health counselor.  Figure 4(a) shows the relationship between attendance and weight loss for months 1 through 9 and at one year. At each time point, greater attendance was significantly associated with greater weight loss. Moreover, there was a significant curvilinear relationship between attendance and weight loss, which became progressively greater over time. This meant that the difference between low and high attendees became greater over time, such that at 40% attendance, weight loss plateaued after 4 months, whereas at 80% attendance, weight loss continued longer into the year. Those meeting with their health counselor nearly every week showed the greatest total weight loss and the longest period of active weight loss.

This is further illustrated by Figure 4(b) showing average weight loss in those who made at least three-quarters of the weekly appointments with their healthcare counselors (high attendees) vs. those who made less than half (low attendees). The high attendees had approximately twice the weight loss as low attendees at three (females: 8.83 ± 0.04 vs. 4.97 ± 0.21; males: 11.9 ± 0.14 vs. 6.84 ± 0.56 kg), six (females: 13.13 ± 0.11 vs. 6.21 ± 0.27; males: 16.51 ± 0.31 vs. 10.09 ± 0.71 kg), nine (females: 15.42 ± 0.21 vs. 6.32 ± 0.25; males: 18.61 ± 0.54 vs. 10.20 ± 0.95 kg), and twelve months (females: 16.49 ± 0.31 vs. 6.03 ± 0.23; males: 19.35 ± 0.81 vs. 8.39 ± 0.67 kg). High attendance was associated with somewhat greater baseline BMI than low attendance (31.73 ± 0.04 vs. 30.92 ± 0.06 kg/m^2^), but little difference in age (52.0 ± 0.09 vs. 52.8 ± 0.12 years) or sex (84.0 ± 0.3% vs. 85.0 ± 0.4% female).

### 3.4. Last Observation Carried Forward (LOCF)

JumpstartMD LOCF weight loss estimates were 6.51 ± 0.03 kg, 7.21 ± 0.03%, and 2.35 ± 0.01 kg/m^2^, with 65.9 ± 0.32% attaining ≥5% and 27.1 ± 0.30% attaining ≥10% weight loss after 13 weeks; 7.70 ± 0.04 kg, 8.45 ± 0.04%, and 2.79 ± 0.01 kg/m^2^, with 66.7 ± 0.32% achieving ≥5% and 36.6 ± 0.33% attaining ≥10% weight loss after 26 weeks; 7.84 ± 0.05 kg, 8.56 ± 0.05%, and 2.84 ± 0.02 kg/m^2^, with 65.7 ± 0.32% achieving ≥5% weight and 36.3 ± 0.33% attaining ≥10% weight loss after 39 weeks; and 7.67 ± 0.05 kg, 8.36 ± 0.05%, and 2.78 ± 0.02 kg/m^2^, with 64.5 ± 0.32% attaining ≥5% and 34.6 ± 0.32% attaining ≥10% weight loss after 52 weeks. Regression analysis of LOCF showed that during the first 3, 6, 9, and 12 months, weight loss averaged (±SE) 1.69 ± 0.08, 1.46 ± 0.11, 1.29 ± 0.12, and 1.17 ± 0.12 kg less in women than men, respectively; 0.007 ± 0.002, 0.020 ± 0.003, 0.023 ± 0.004, and 0.025 ± 0.004 kg more per year of age, respectively; and 0.24 ± 0.01, 0.36 ± 0.0.01, 0.41 ± 0.01 and 0.42 ± 0.01 kg more per increment in baseline BMI, respectively. These analyses represent the independent effect of each factor. The male-female difference increased when adjusted for enrollment duration and attendance (i.e., 1.89 ± 0.06 kg greater weight loss in men than women at 13 weeks, 1.90 ± 0.08 kg at 26, 1.84 ± 0.10 kg at 39 weeks, and 1.72 ± 0.10 kg at 52 weeks).

### 3.5. Weight Loss Drugs

Phentermine and phendimetrazine showed little effect on weight loss in completers except in women during the first two months of treatment. Patients prescribed these drugs were more likely to be females (23.7%) than males (17.4%), younger (prescribed vs. non-prescribed mean ± SE, female: 51.0 ± 0.17 vs. 52.0 ± 0.10; male: 51.0 ± 0.46 vs. 54.2 ± 0.23 years), and were slightly leaner if female (30.82 ± 0.08 vs. 31.29 ± 0.05 kg/m^2^, *P*=1.2 × 10^−6^) but not male (34.31 ± 0.22 vs. 34.26 ± 0.10 kg/m^2^). Among female completers, those prescribed phentermine or phendimetrazine lost 6.1% more weight by the end of the first month (difference±SE: −0.24 ± 0.04 kg, *P*=2.2 × 10^−8^) and 4.1% more weight by the end of the second month (−0.26 ± 0.06 kg, *P*=4.9 × 10^−5^), but only 1.2% more weight loss after 3 months (−0.10 ± 0.09 kg, nonsignificant), and no significant weight loss advantage thereafter. However, retention was greater in those prescribed weight loss drugs. Specifically, the pharmacologically treated patients showed longer average enrollment than the untreated during the first three (females: 2.51 ± 0.01 vs. 2.14 ± 0.01; males: 2.52 ± 0.03 vs. 2.20 ± 0.02 months), six (females: 4.49 ± 0.03 vs. 3.41 ± 0.02; males: 4.48 ± 0.07 vs. 3.50 ± 0.04 months), nine (females: 6.18 ± 0.04 vs. 4.31 ± 0.03; males: 6.10 ± 0.12 vs. 4.37 ± 0.04 months), and twelve months (females: 7.73 ± 0.06 vs. 5.07 ± 0.03; males: 7.48 ± 0.16 vs. 5.08 ± 0.07 months). Correspondingly, those prescribed drugs showed greater LOCF weight loss after three (females: 7.01 ± 0.06 vs. 5.83 ± 0.04; males: 9.18 ± 0.35 vs. 8.43 ± 0.11 kg), six (females: 8.66 ± 0.09 vs. 6.84 ± 0.05; males: 11.15 ± 0.41 vs. 9.64 ± 0.14 kg), nine (females: 8.98 ± 0.10 vs. 6.93 ± 0.05; males: 11.19 ± 0.42 vs. 9.79 ± 0.15 kg), and 12 months than nonusers (females: 8.81 ± 0.11 vs. 6.78 ± 0.05 kg; males: 10.73 ± 0.42 vs. 9.59 ± 0.15 kg). Adjustment for enrollment eliminated the LOCF weight loss difference between drug users and nonusers after three (unadjusted vs. adjusted difference ± SE, female: −1.18 ± 0.07 difference reduced to −0.18 ± 0.06 kg difference when adjusted, male: −0.76 ± 0.29 reduced to +0.46 ± 0.25 kg), six (female: −1.83 ± 0.10 reduced to −0.03 ± 0.08 kg, male: −1.51 ± 0.36 reduced to +0.57 ± 0.31 kg), nine (female: −2.05 ± 0.11 reduced −0.02 ± 0.10 kg, male: −1.40 ± 0.38 reduced to +1.00 ± 0.34 kg), and twelve months (female: −2.02 ± 0.11 reduced to −0.02 ± 0.10 kg, male: −1.13 ± 0.38 reduced to +0.99 ± 0.36 kg).

### 3.6. Weight Loss Prediction

Several studies suggest that rapid initial weight loss predicts longer-term weight loss [20–22, 26, 62]. Weight loss during the first 2 weeks was significantly correlated with LOCF weight loss at three (*r* = 0.62), six (*r* = 0.52), nine (*r* = 0.49), and twelve months (*r* = 0.47).

### 3.7. Comparison of LOCF with Other Commercial Programs

**Figure 5:**
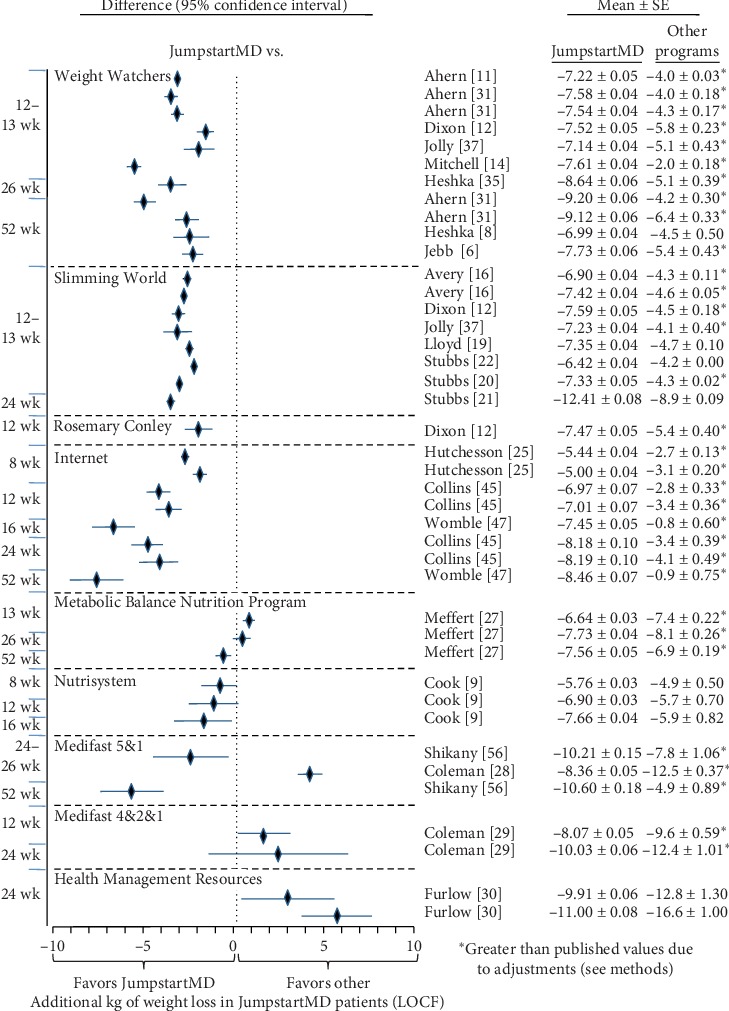
Mean LOCF weight loss difference between JumpstartMD and other commercial programs as measured by Δkg weight loss. Diamonds represent the difference between the adjusted JumpstartMD mean and the other commercial programs, and the horizontal bars represent the corresponding 95% confidence interval for the difference. Negative differences designate greater JumpstartMD weight loss. The time point of the comparison is to the left of the plot. ^*∗*^Weight loss in the other commercial programs higher than published value due to adjustment (see Methods). ^†^Mean change ± SE. ^‡^Mean change ± SE_prediction_ adjusted to the recruitment BMI range and baseline age, proportion of males, and sex-specific BMI effects of the commercial study.

**Figure 6:**
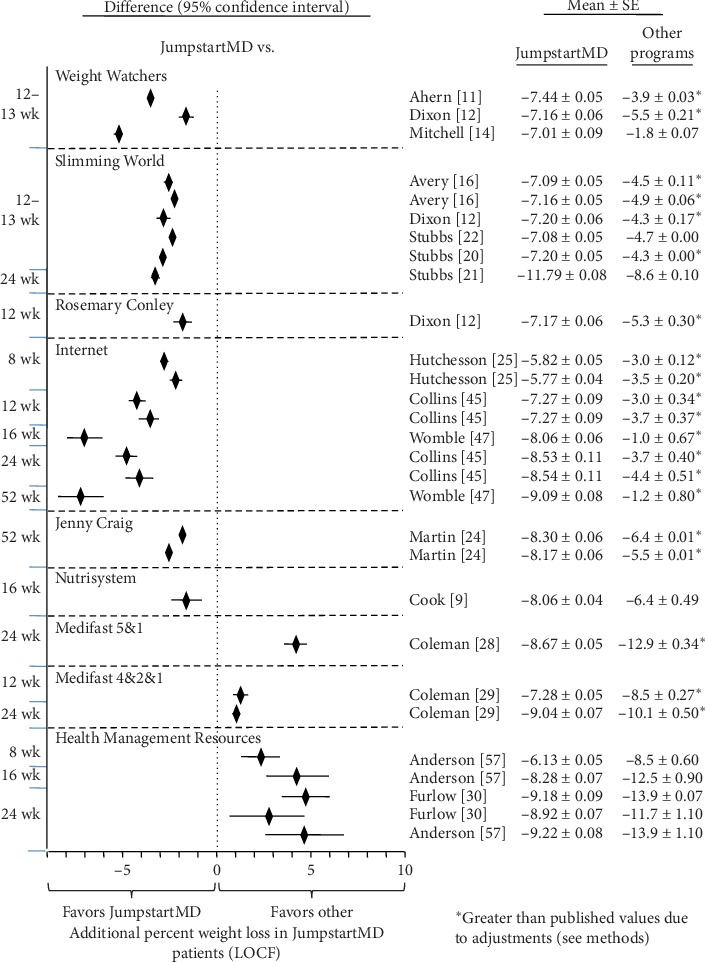
Mean LOCF weight loss difference between JumpstartMD and other commercial programs as measured by %weight loss. Negative differences designate greater JumpstartMD %weight loss. See legend of Figure 5 for further explanation.

**Figure 7:**
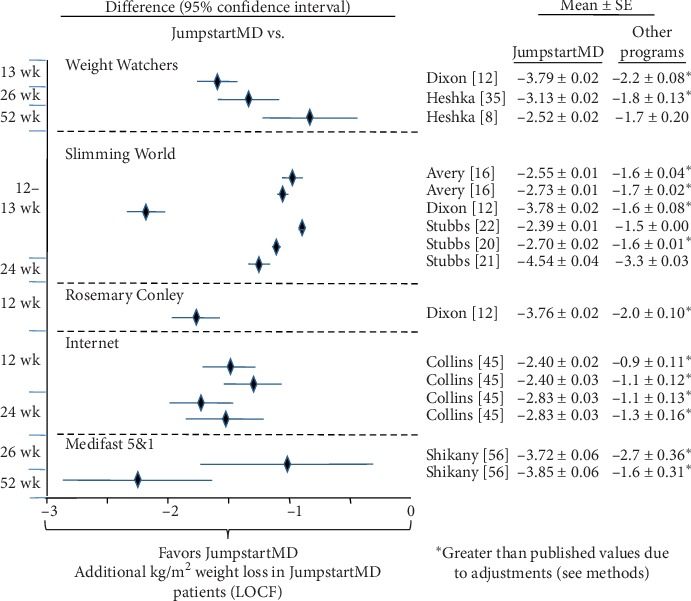
Mean LOCF weight loss difference between JumpstartMD and other commercial programs as measured by ΔBMI. Negative differences designate greater JumpstartMD BMI loss. See legend to Figure 5 for further explanation.

**Figure 8:**
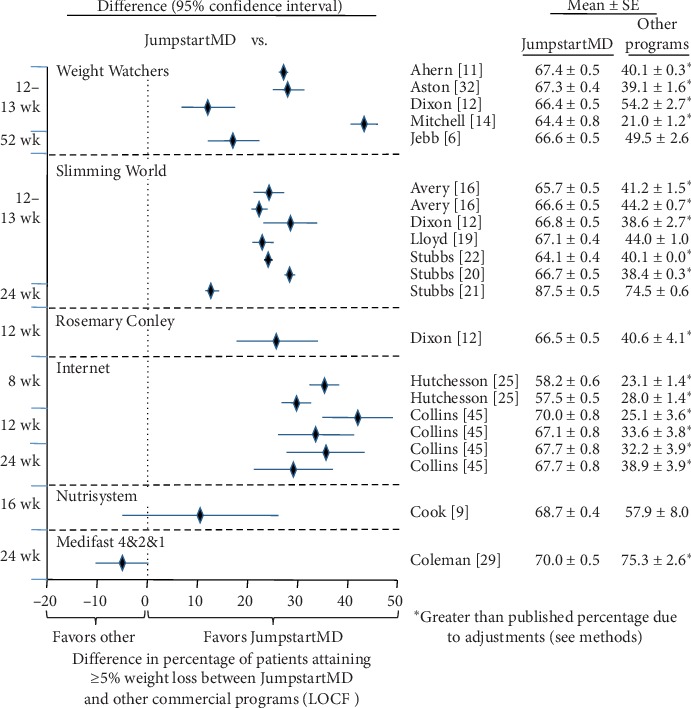
Mean LOCF weight loss difference between JumpstartMD and other commercial programs as measured by percent attaining ≥5% weight loss. Positive differences designate greater JumpstartMD weight loss. See legend to Figure 5 for further explanation.

**Figure 9:**
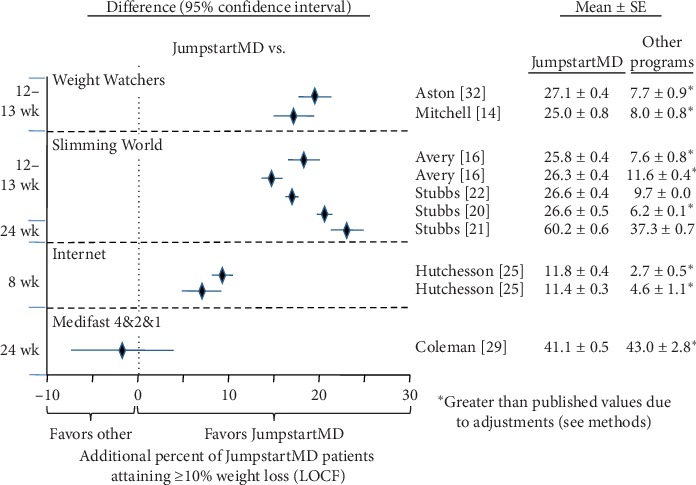
Mean LOCF weight loss difference between JumpstartMD and other commercial programs as measured by percent attaining ≥10% weight loss. Positive differences designate greater JumpstartMD weight loss. See legend to Figure 5 for further explanation.

**Figure 10:**
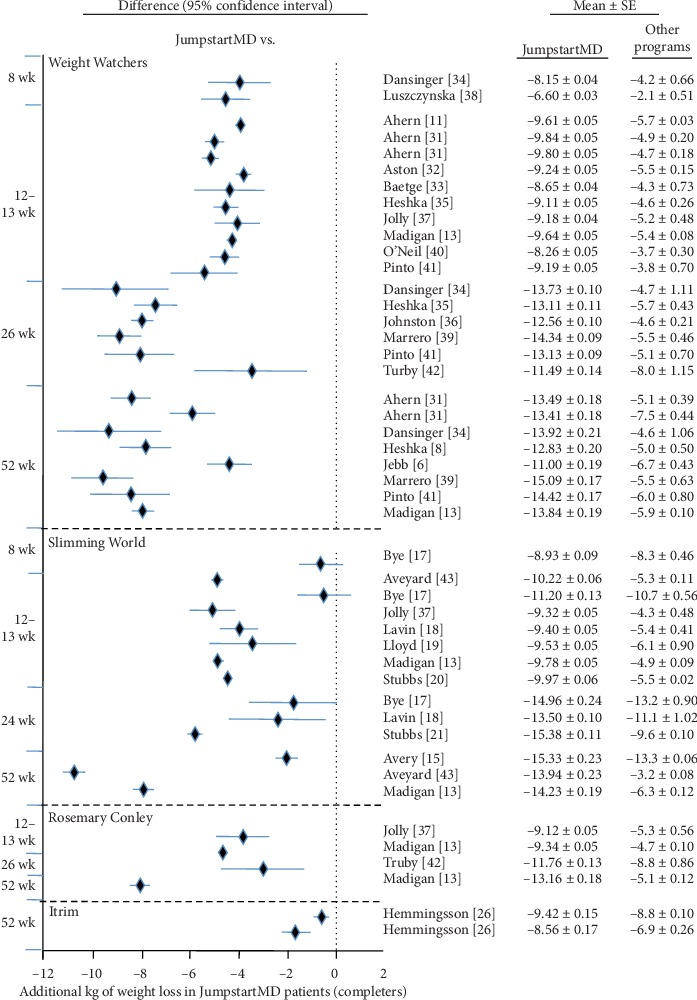
Mean weight loss difference between JumpstartMD and other commercial programs in completers as measured by kg weight loss. Negative differences designate greater JumpstartMD weight loss. See legend to Figure 5 for further explanation.

**Figure 11:**
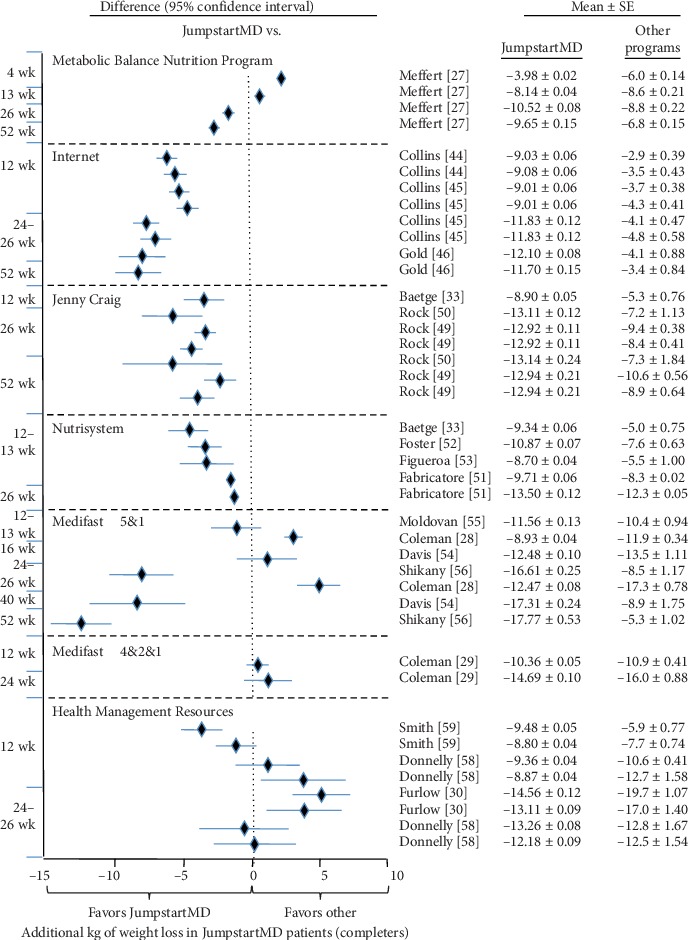
Mean weight loss difference between JumpstartMD and other commercial programs in completers as measured by kg weight loss (continued). Negative differences designate greater JumpstartMD weight loss. See legend to Figure 5 for further explanation.

**Figure 12:**
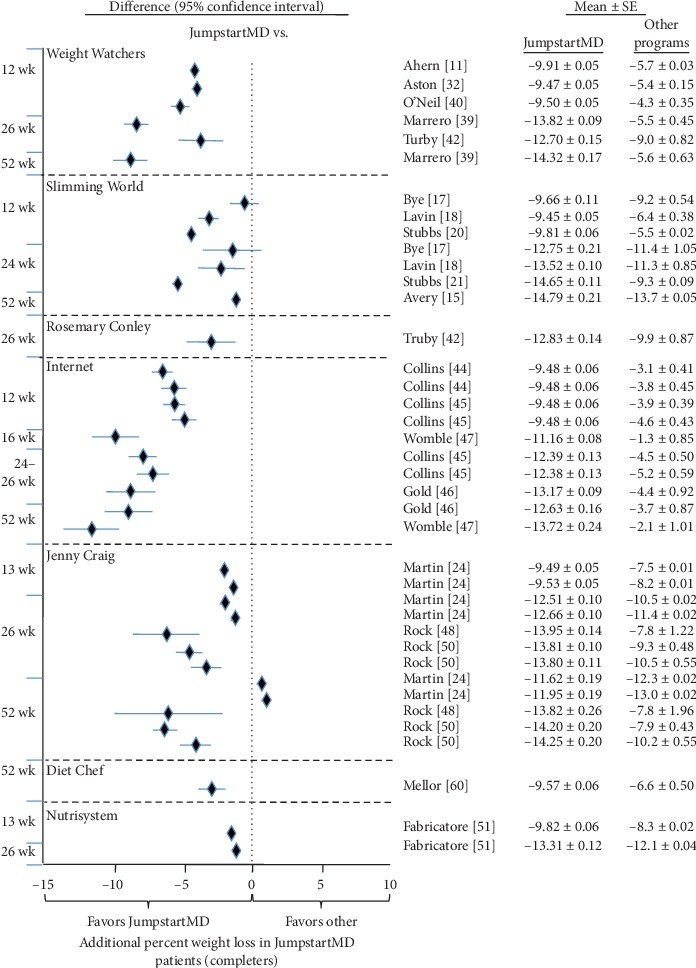
Mean weight loss difference between JumpstartMD and other commercial programs in completers as measured by percent weight loss. Negative differences designate greater JumpstartMD %weight loss. See legend to Figure 5 for further explanation.

**Figure 13:**
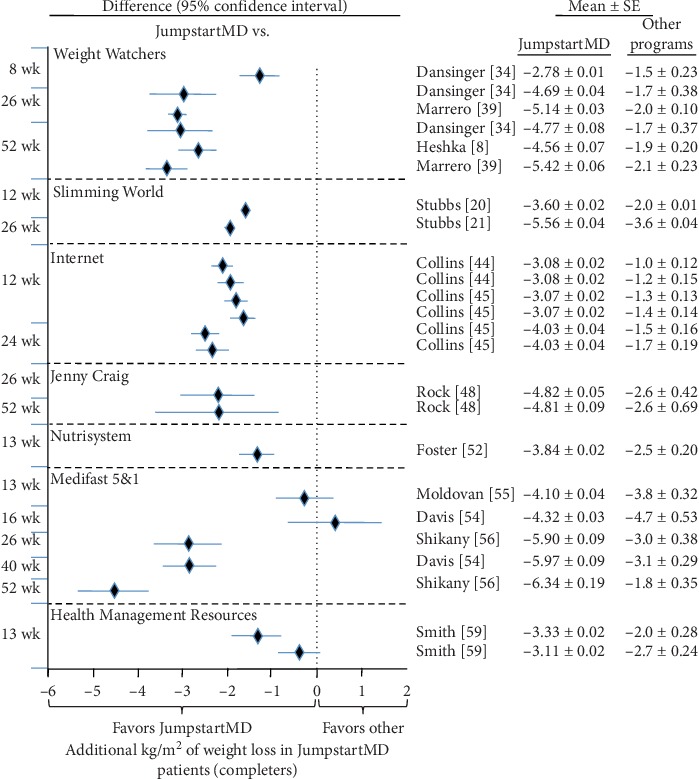
Mean weight loss difference between JumpstartMD and other commercial programs in completers as measured by ΔBMI. Negative differences designate greater JumpstartMD BMI loss. See legend to Figure 5 for further explanation.

**Figure 14:**
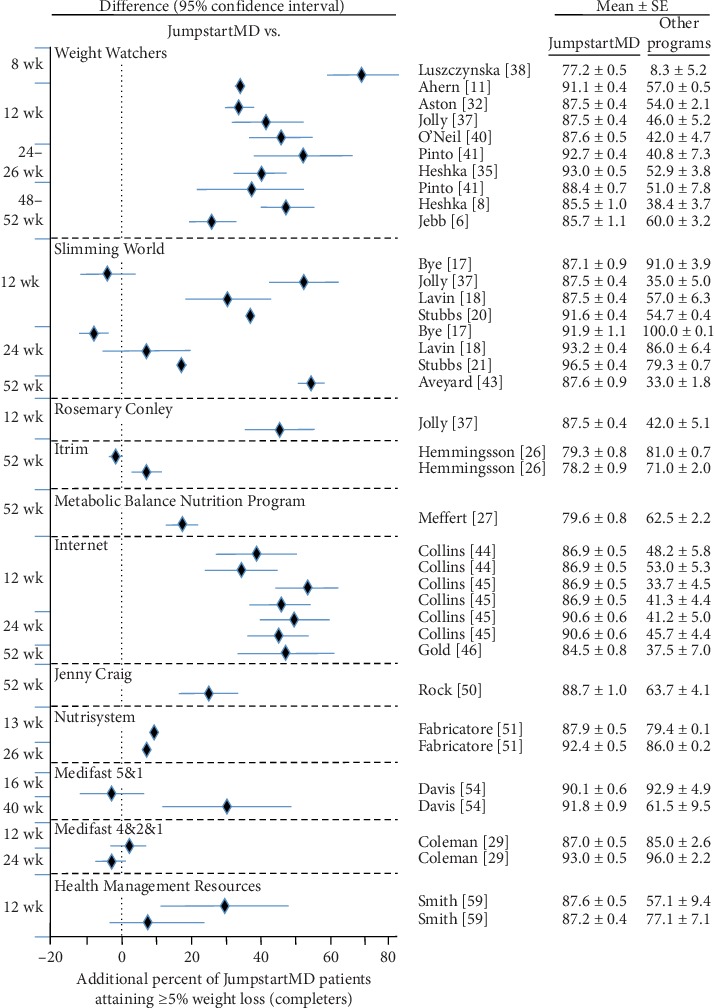
Mean weight loss difference between JumpstartMD and other commercial programs in completers as measured by percent attaining ≥5% weight loss. Positive differences designate greater JumpstartMD weight loss. See legend to Figure 5 for further explanation.

**Figure 15:**
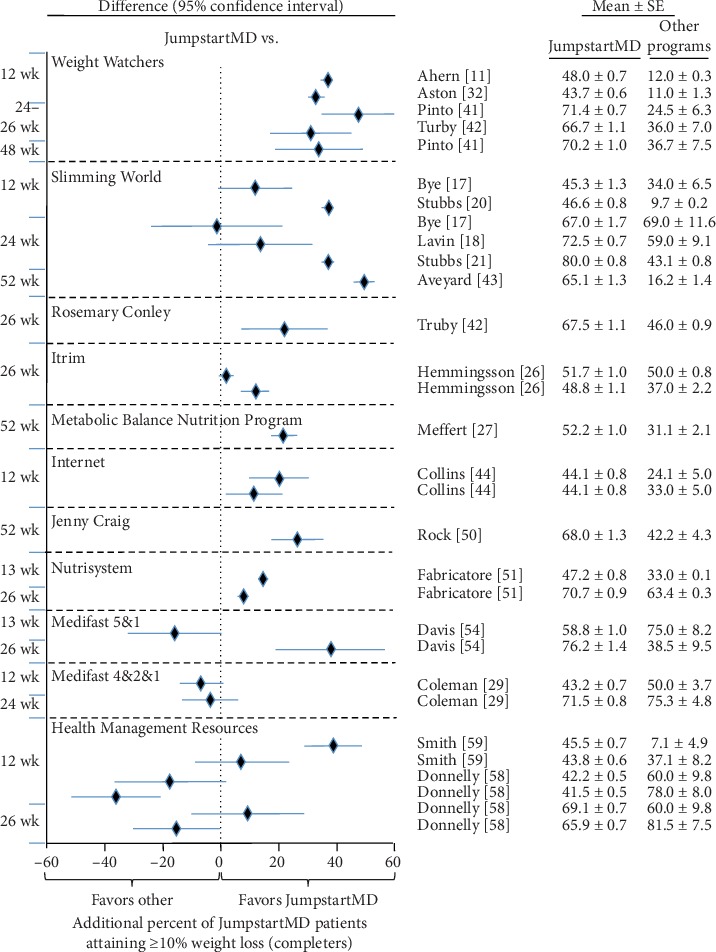
Mean weight loss difference between JumpstartMD and other commercial programs in completers as measured by percent attaining ≥10% weight loss. Positive differences designate greater JumpstartMD weight loss. See legend to Figure 5 for further explanation.

Figures 5through 15 present the individual weight loss differences between JumpstartMD and other commercial studies when matched for follow-up duration, recruitment BMI, baseline age, proportion of males, and sex-specific baseline BMI effects. Negative differences for Δweight loss, Δ% weight loss, and ΔBMI represent greater weight loss in JumpstartMD patients. The average fixed effect meta-analysis estimates of the weight loss differences between JumpstartMD and these other programs are presented in Table 3. JumpstartMD weight losses were significantly greater than the average published losses over all calorie/food/meal plan (Weight Watchers, Slimming World, Rosemary Conley, and Itrim), and Internet programs (Biggest Loser Club) for all measures of weight loss (Table 3, Figures 5–15).

**Figure 16:**
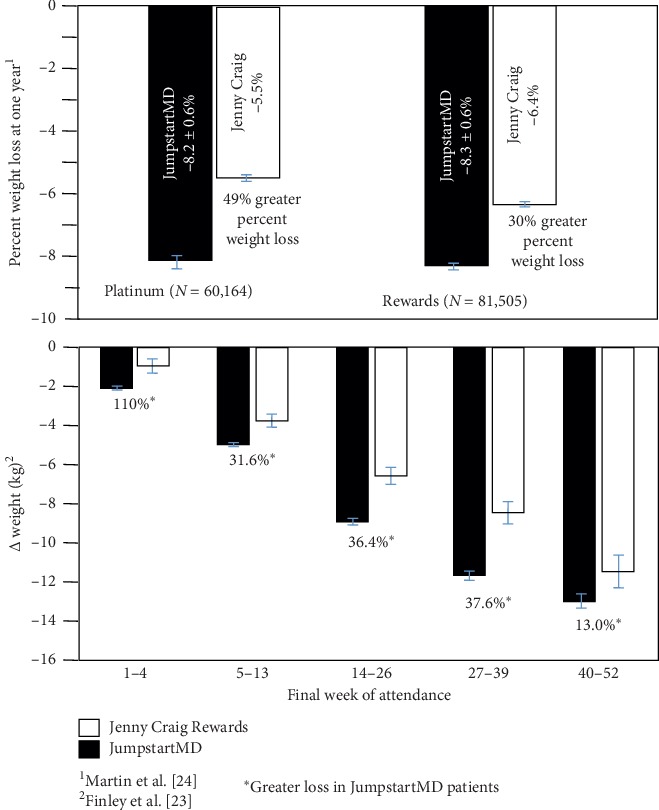
Mean LOCF percent weight loss difference between JumpstartMD and the published Jenny Craig weight losses by Martin et al. (upper panel) [24] and Finley et al. (lower panel) [23]. JumpstartMD samples were selected to correspond to the Jenny Craig analyses.

Except for percent weight loss at 52 weeks, JumpstartMD weight loss was also greater than those reported for prepackaged meals (Table 3).  Figure 16 (top panel) shows that one-year LOCF percent weight loss was greater for JumpstartMD than Jenny Craig self-paying customers for their Platinum (49% greater percent weight loss) and Rewards program (30% greater [24]). They also reported the Rewards program percent weight loss by final week of attendance [23]. JumpstartMD LOCF weight loss was over 30% greater through the 39th week and 13% greater between weeks 40 and 52.

JumpstartMD weight loss was almost always greater than those reported for meal replacement programs in completers due to the inclusion of a very large sample of Nutrisystem completers [51]. JumpstartMD kg weight loss was 18% greater than reported for 103,693 Nutrisystem patients at 3 months and 9% greater than reported for 32,280 patients at 6 months (Figure 11). These data are not included in the LOCF analyses or the completer's analysis at 1 year. The figures suggest that Medifast 5&1 plans produced significantly less weight loss than JumpstartMD in the study by Shikany et al. [56], greater weight loss in the study by Coleman et al. [28], and mixed results in the study by Davis et al. [54]. Medifast 4&2&1 plan and Health Management Resources (combination of meal replacement and prepackaged meals) showed significantly greater weight loss than JumpstartMD.

## 4. Discussion

Although eighty percent of weight loss attempts are self-directed [63], commercial weight loss programs provide valuable consumer options for those desiring support. Our analyses of completers show that a medically supervised real-food, low-calorie, low-carbohydrate diet provided through a one-on-one in person behavioral intervention produced substantial average weight losses after 3 (8.7 kg or 9.5%), 6 (11.8 kg or 12.6%), and 12 months (11.5 kg or 12%). Five percent weight loss was achieved by 86.3% of JumpstartMD patients after 3 months, 90% after six months, and 81% after one year, over twice the weight loss identified by the FDA as an effective product (thirty-five percent achieving ≥5% weight loss [64]). Even greater average weight losses were attained in patients who attended at least 75% of their week appointments with their personal healthcare coach after 3 (women: 8.8 kg; men: 11.9 kg), 6 (women: 13.1 kg; men: 16.5 kg), and 12 months (women: 16.5 kg; men: 19.35 kg). Consistent with other reports [42], the weight loss tended to be rapid initially, diminishing thereafter, and then plateau as compensatory mechanisms that tend to increase food intake and reduce weight loss kick in. However, Figure 4 showed weight loss continued to be accrued throughout the year at higher participation levels. This is consistent with other reports showing the importance of patient involvement [8, 11, 14, 19–22, 27, 28, 32, 34–36, 40, 41, 45, 46].

The JumpstartMD results are consistent with other studies showing age [11, 19], sex [11, 12, 15, 19–22, 29, 30], and initial body weight [11, 12, 20, 21, 26, 42] affect weight loss success. The greater weight loss in heavier subjects may account in part for the greater weight loss success in men given that the sex difference largely disappeared for percent weight loss (Figure 2).

A one-on-one physician-supervised program that provides individual health counselors and nutritional advice must necessarily be more expensive than group-based and Internet-based therapies, and this additional cost warrants scrutiny in terms of weight loss success. Moreover, although important health benefits accrue for 5% or 10% weight loss, those seeking treatment frequently aspire to ≥25% weight loss and are more likely motivated by appearance rather than health [65].

Our comparative analyses were restricted to the intervention arm of the randomized trial (representing only 0.3% of the published patient population) and longitudinal patient data (99.7% of the published patient population) because we believe these uncontrolled values accurately reflect the treatment effects. Specifically, in the absence of illness or behavioral change, large cohorts of individuals do not lose weight over time, with most Western adults gaining 0.2 to 2.0 kg per annum [66, 67]. None of the wait-listed or usual diet control arms of commercial programs RCT show any significant weight loss, i.e., weight change from baseline: −0.3 ± 0.35 kg [59], +0.16 ± 0.88 kg [33], +0.84 ± 0.41 kg [42], +0.36 ± 0.24 kg [44], −0.25 kg [58], and −0.6 ± 0.71 kg [52]. In addition, clinical trial participants are not representative of the general population, do not pay for their own treatment (selection bias), and are subtly coerced to participate and not dropout (performance bias). Hence, the call for “naturalistic studies” of large cohort followed prospectively that report retention rates and weight loss at discontinuation [68].

With one notable exception, the comparative analyses of Table 3 show that JumpstartMD patients lost greater weight than reported for other calorie-counting, food choice, and meal plans (Weight Watchers, Slimming World, Rosemary Conley, and Itrim). JumpstartMD kg weight loss was 53% greater at 12-13 weeks, 84% greater at 24–26 weeks, and 75% greater at 1 year than reported for other commercial calorie-counting programs for LOCF analyses and approximately 2-fold greater in completers analyses. Compared to Internet-based commercial programs (Biggest Losers Club, E-diet), JumpstartMD weight loss averaged approximately 1.5-fold greater for LOCF and 1.75-fold greater for completers. The notable exception is the Metabolic Balance Nutrition Program [27], a program similar to JumpstartMD in providing low-carbohydrate diet through individualized food lists and meal plans and individual support by certified advisors. We interpret the consistent weight losses reported for JumpstartMD in this report and the Metabolic Balance Nutrition Program as evidence that our approach is replicable across programs.

Jenny Craig (Jenny Craig, Inc; Carlsbad, CA) uses individual counseling, 1200–2000 kcal/d low-energy density diet, prepackaged foods that are gradually replaced with normal foods, and increased physical activity to promote weight loss. When the data were analyzed by last week of attendance, the JumpstartMD patients lost more kg weight than the 60,164 Platinum patients during weeks 1–4 (110% more), 5–13 (32%), 14–26 (36%), 27–39 (38%), and weeks 40–52 (13% more) [23]. JumpstartMD % weight losses were 49% greater than the LOCF weight losses reported by Martin et al. for the Platinum Plan and 30% greater than the more current Rewards Plan [24] (Figure 16).

The largest meal replacement program in the United States is Nutrisystem. JumpstartMD weight loss was 18% to 30% greater than that reported for Nutrisystem by Cook et al. [9]. A poster presentation on completers by Fabricatore et al. reported on 103,693 self-reported weights at 3 months and 32,280 self-reported weights at 6 months [51]. JumpstartMD kg weight loss was 18% greater at 3 months and 9% greater after 6 months (Figure 11); however, these percentages may underestimate the true differences given that unsuccessful weight loss would likely discourage participation in the Nutrisystem optional online website. Indeed, Johnston et al. reported that Weight Watcher patients who made greater use of their online website were 3.1-fold more likely to reach 5% weight loss after 6 months and 5.4-fold more likely to achieve 10% weight loss than low-use patients [36]. We expect this probably largely reflects self-selection of high-adherence to the Weight Watchers program given that simply recording weight produces minimal weight loss [25, 44, 45, 47]. JumpstartMD weight losses were somewhat smaller than those reported by Coleman et al. for Medifast [28, 29] and by Furlow et al. for Health Management Resources [30].

### 4.1. Pharmacotherapy

Unexpectedly, phentermine and phendimetrazine had, at best, a modest weight loss effect in the context of the low-calorie low-carbohydrate diet and behavioral intervention, perhaps because it is not a central part of the medically supervised lifestyle modification program and not central to a participant's success. Its principal benefit was prolonging exposure to the weight loss intervention leading to additional weight loss consistent with nonpharmacologically treated patients.

### 4.2. Limitations

Although participants tracked their diets using supplied forms or their own mobile phone software and shared this information with their health coach, these data were not collected for data analysis. Thus, there is no statistical verification of their carbohydrate intake or dietary caloric deficit. The superiority of JumpstartMD vis-à-vis other commercial programs does not exclude the possibility that patients choosing JumpstartMD may be more predisposed to weight loss (i.e., self-selection). Comparisons between JumpstartMD and other programs may be somewhat skewed by the fact that JumpstartMD participants could afford to pay $350 to $400 per month and the costs associated with a healthy low-carbohydrate diet and physical activity. Monthly pricing often diminished after 4 months on program because members had an option to transition from a weekly to a twice monthly for weight loss or maintenance or later for a monthly visit structure at a lower monthly fee for maintenance only. We do not have information on the income levels or health literacy of the JumpstartMD population, which could contribute to weight loss differences of the figures. The data available for statistical analyses also lacked detailed medical record information; however, medical histories were collected and reviewed by the clinical staff and adjustment made to the dietary prescription as appropriate. Although a low-carbohydrate intake was generally prescribed, the exact carbohydrate goals varied by participant in accordance with their weekly clinical assessments and interviews to judge the patient's tolerance to the diet. Eliminating the possible effects of self-selection would require a randomized trial, an approach that has largely failed to distinguish weight loss differences between other commercial programs [33, 37, 42, 68]. We also caution that these analyses rely on previously published reports, which may not necessarily represent current practices in other commercial programs.

### 4.3. Conclusions

JumpstartMD appears to provide greater weight loss than most other published commercial programs (i.e., Weight Watchers, Slimming World, Rosemary Conley, Jenny Craig, and Nutrisystem), presumably because it provides resources necessary for comprehensive individualized nutritional and behavioral counseling in a medically supervised environment and because the low-carbohydrate dietary approach may promote greater satiety and voluntary limitation of caloric intake [69]. Increased protein intake associated with a low-carbohydrate diet may also have contributed to increased satiety [70]. Accessibility to the JumpstartMD program is dependent upon financial resources. It addresses the weight loss needs of those patients requiring or desiring greater personal attention than provided in less-expensive group instruction or online programs. Commercial weight loss programs offer a spectrum of personal support. Programs like Weight Watchers and Slimming World would be more easily scaled to address the weight loss needs of sizable overweight population at lower socioeconomic status. In addition, programs such as Weight Watchers may provide more pounds lost per dollar spent [71]. However, JumpstartMD may be a better option for those valuing high touch, individualized attention and greater total weight loss combined with the associated health and aesthetic benefits. Moreover, pounds per dollar spent may be too narrow a criterion given that obese patients incur 46% higher inpatient costs, 27% more physician visits and outpatient costs, and 80% higher prescription costs than normal weight individuals [72].

## Figures and Tables

**Table 1:** Sample characteristics of studies on commercial weight loss programs.

Study	Description	Selection criteria		Recruited sample		
		Age^*∗*^	BMI	Age	% male	BMI^*∗*^
*Weight Watchers (WW)*						
Ahern et al. [11]	NHS referral to 12 meetings 4/2/07–10/6/09 (*N* = 29,326)	Adult		49 (17)	10	35.1 (5.7)
Ahern et al. [31]	RT of 12 wk WW vs. self-help. 3 mo and 12 mo endpoints.	>18	≥28	53.6 (13.3)	32	34.7 (5.4)
Ahern et al. [31]	RT 52 of wk WW (*N* = 528) vs. self-help	≥18	≥28	53.3 (14.0)	32	34.5 (5.1)
Aston et al. [32]	NHS referral 6/28/05 to 5/30/07 (*N* = 967)	16–81	≥30	49.4 (8.5)	12.3	35.2 (6.4)
Baetge et al. [33]	RT of WW (*N* = 29) vs. others. 12 wk.	18–69	27–50	48 (11)	0	34 (6)
Dansinger et al. [34]	RT of WW (*N* = 40) vs. others	22–72	27–42	49 (10)	42	35 (3.8)
Dixon et al. [12]	North Somerset NHS referral 12/01/07–5/31/10 (*N* = 414) 12 wk.	≥16	≥30	46.8 (15.0)	11.5	37.9 (6.2)
Heshka et al. [35]	RT of WW (*N* = 211) vs. self-help. 26 wk	18–65	27–40	45 (10)	18	33.8 (3.4)
Heshka et al. [8]	RT of WW (*N* = 211) vs. self-help. 1 and 2 yr results	18–65	27–40	45 (10)	18	33.8 (3.4)
Jebb et al. [6]	RT of WW (*N* = 377) vs. standard care	≥18	27–35	46.5 (13.5)	12	31.5 (2.6)
Johnston et al. [36]	RT of WW (*N* = 147) vs. self-help	≥18	27–40	47.5 (11.7)	10.9	33.1 (3.7)
Jolly et al. [37]	RT of WW (*N* = 100) vs. other after 12 wk	≥18	≥30	50.7 (14.6)	28	34.0 (3.9)
Luszczynska et al. [38]	RT of WW (*N* = 55) assigned to implementation intention prompts or control.	18–76	25.3–48.3	44.0 (14.0)	0	33.4 (6.5)
Madigan et al. [13]	South Birmingham NHS referral 5/01/09–3/31/10 (*N* = 1366) for 13 wk	≥18	≥28	48.9 (15.2)	12.2	35.2 (5.8)
Marrero et al. [39]	RT of WW (*N* = 112) vs. diabetes prevention program	≥18	≥24	51.5 (11.5)	17	36.9 (7.3)
Mitchell et al. [14]	Tennessee Medicaid WW referrals in 2006 (*N* = 1192)	≥10	Mostly > 30	34.9	3.6	39.6
O'Neil et al. [40]	RT to two methods for calculating and limiting food intake (*N* = 111)	25–65	27–35	49.7 (10.9)	10.8	31.5 (2.2)
Pinto et al. [41]	RT of WW (*N* = 49) vs. behavioral intervention. 48 wk	30–65	27–50	49.0 (9.2)	10.2	35.5 (5.3)
Truby et al. [42]	RT of WW (*N* = 58), vs. other	18–65	27–40	39.9 (10.9)	27.6	31.2 (2.7)

*Slimming World (SW)*						
Avery et al. [15]	Enrolled 1/1/2012–3/31/2012 (*N* = 24,447) for ≥1 year	≥18	≥30	47.6 (13.7)	8.3	37.1 (5.9)
Avery and Morris [16]	Referred to SW by general practitioner (*N* = 5482)	≥18	≥25	48.8 (14.7)	13.2	37.3 (6.4)
Avery et al. [16]	Referred to SW by Healthy Living Pharmacy (*N* = 1020)	≥18	≥25	43.5 (13.9)	8.9	35.1 (6.3)
Aveyard et al. [43]	RT of primarily SW (*N* = 940) vs. advice only.	≥18	Mostly ≥30	56	42.7	34.8 (4.6)
Bye et al. [17]	7 of 13 men-only groups opened 6–9 mo (*N* = 67).	29–71	27.9–53.6	47	100	35.9
Dixon et al. [12]	North Somerset NHS referral 12/01/07–5/31/10 (*N* = 450)	≥16	≥30	47.7 (14.5)	16.8	37.7 (5.8)
Jolly et al. [37]	RT of SW (*N* = 100) vs. other after 12 wk	≥18	≥30	48.8 (14.9)	35	33.8 (3.8)
Lavin et al. [18]	General practice patients (*N* = 107)	23–78	30–47	49.5	11	36
Lloyd and Khan [19]	Dorset, UK referrals from 10/1/08–9/30/09 (*N* = 2456)	>18	≥28	51.1 (15.0)	13	36.8 (6.3)
Madigan et al. [13]	South Birmingham Primary Care Trust NHS referral 5/01/09–3/31/10 (*N* = 921) for 13 wk	≥18	≥28	49.6 (14.5)	13.4	35.7 (6.1)
Stubbs et al. [20]	Primary Care Trust referral from 5/1/2004–11/30/2009 (*N* = 34,271)	≥16	Mostly ≥30	47.3 (14.4)	10.7	36.8 (6.5)
Stubbs et al. [21]	NHS 24 wk referrals 5/1/2004–11/30/2009 (*N* = 4754)	≥16	Mostly ≥30	49.8 (14.3)	12.1	37.9 (6.7)
Stubbs et al. [22]	Self-referred patients 1/1/2010–4/30/2012 (*N* = 1,356,105)	19–79	20–90	42.3 (13.6)	5	32.6 (6.3)

*Rosemary Conley (RC)*						
Dixon et al. [12]	North Somerset NHS referrals 12/01/07–5/31/10 (*N* = 143)	≥16	≥30	46.7 (13.5)	11.2	37.6 (5.7)
Jolly et al. [37]	RT of RC (*N* = 100) vs. other after 12 weeks	≥18	≥30	49.8 (14.5)	31	33.4 (3.5)
Madigan et al. [13]	South Birmingham NHS referrals 5/01/09–3/31/10 (*N* = 791) for 13 weeks	≥18	≥28	50.1 (14.4)	11.5	34.3 (5.1)
Truby et al. [42]	RT of RC (*N* = 58) vs. other	18–65	27–40	40.6 (10.3)	27.6	31.6 (2.6)

*Biggest losers club*						
Collins et al. [44]	RT of basic website (*N* = 99) vs. control. at 12 wk	18–60	25–40	42.0 (10.9)	41	32.3 (3.6)
Collins et al. [44]	RT of enhanced website (*N* = 106) vs. control at 12 wk	18–60	25–40	42.2 (10.2)	42	32.3 (4.3)
Collins et al. [45]	RT of basic website (*N* = 143) vs. others at 24 wk	18–60	25–40	41.9 (10.1)	47.2	32.2 (3.7)
Collins et al. [45]	RT of enhanced website (*N* = 158) vs. others. 24 wk	18–60	25–40	42.0 (10.3)	52.8	32.2 (4.1)
Hutchesson et al. [25]	Regular subscribers 6/1/2011–10/24/2011 (*N* = 953)		>18.5	36.5 (10.7)	14.8	33.0 (7.1)
Hutchesson et al. [25]	Fast-track subscribers 6/1/2011–10/24/2011 (*N* = 381)		>18.5	37.1 (9.1)	12.3	30.6 (6.5)

*eDiet*						
Gold et al. [46]	RT of E-diet (*N* = 62) vs. others	>18	25–40	48.9 (9.9)	15	32.5 (4.2)
Womble et al. [47]	RT of E-diet (*N* = 23) vs. others	18–65	27–40	44.2 (9.3)	0	33.9 (3.2)

*Jenny Craig (JC)*						
Baetge et al. [33]	RT of JC (*N* = 27) vs. other	18–69	27–50	46 (12)	0	35 (5)
Finley et al. [23]	Platinum JC enrollees between May 2001 and May 2002 (*N* = 60,154)	18–79		43.2	8	89.9 kg
Martin et al. [24]	Platinum JC enrollees 5/1/2001–5/1/2002 (*N* = 60,164)	18–75		43.2	8	89.9 kg
Martin et al. [24]	Improved JC program 1/1/2005–12/31/2005 (*N* = 81,505)	18–75		43.7	10.3	92.2 kg
Rock et al. [48]	RT of JC (*N* = 35) vs. control	≥18	25–40	42 (11)	0	34.2 (3.7)
Rock et al. [49]	RT of JC with center nutritional counseling (*N* = 167) vs. others	18–69	25–40	44 (10)	0	33.8
Rock et al. [49]	RT of JC with telephone nutritional counseling (*N* = 164) vs. others	18–69	25–40	44 (10)	0	33.8
Rock et al. [50]	RT of low-fat JC version (*N* = 74) vs. control in type 2 diabetics	≥18	25–45	55.5 (9.2)	52.7	36.2 (4.3)
Rock et al. [50]	RT of low-carbohydrate JC version (*N* = 77) vs. control in type 2 diabetics	≥18	25–45	57.3 (8.6)	52	36.2 (4.7)

*Itrim*						
Hemmingsson et al. [26]	Meal replacement Itrim enrollees 1/1/2006–5/31/2009 (*N* = 4588)			50 (11)	14	30 (4)
Hemmingsson et al. [26]	Restricted calorie Itrim enrollees 1/1/2006–5/31/2009 (*N* = 676)			51 (12)	19	29 (5)

*Nutrisystem*						
Baetge et al. [33]	RT to Nutrisystems (*N* = 28) vs. other	18–69	27–50	46 (12)	0	37 (5)
Cook et al. [9]	RT of Nutrisystems (*N* = 38) vs. self-directed	18–70	25–45	51.5 (10.9)	21.1	33.5 (4.5)
Fabricatore et al. [51]	Enrollees 1/1/2008–12/31/2010 w 3 mo data (*N* = 103,693)	All	>25	46.9 (12.4)	30.2	34.3 (6.6)
Fabricatore et al. [51]	Enrollees 1/1/2008–12/31/2010 w 6 mo data (*N* = 32,280)	All	>25	48.3 (12.4)	28.8	35.0 (6.9)
Foster et al. [52]	RT of Nutrisystems (*N* = 35) vs. education in type 2 diabetics	21–75	30–50	52.1 (7.7)	25.7	39.1 (5.5)
Figueroa et al. [53]	RT of Nutrisystems (*N* = 13) vs. exercise or Nutrisystems plus exercise	Postmenopause	≥25	54.1 (6)	0	34.8 (4.3)

*Metabolic Balance Nutrition Program*						
Meffert and Gerdes [27]	Prospective cohort (*N* = 524)	19–81		50 (12.0)	15.9	30.3 (5.7)

*Medifast*						
Coleman et al. [28]	3-center retrospective chart review of 5&1 plan from 2007–2010 (*N* = 446)	18–70	≥25	47 (10.1)	13.5	34.3 (6.2)
Coleman et al. [29]	21-center retrospective chart review of 4&2&1 plan from 1/1/2012–3/31/2014 (*N* = 310)	≥18	≥25	53.5 (14.7)	42.9	37.7 (6.8)
Davis et al. [54]	RT of 5&1 plan (*N* = 45) vs. self-selected isocaloric plan	18–65	30–50	43.0 (10.2)	33.3	38.5 (6.8)
Moldovan et al. [55]	RT of 5&1 plan alone (*N* = 38) vs. with phentermine.	35–70	35–50	48.4 (9.4)	23.7	42.7 (8.7)
Shikany et al. [56]	RT of 5&1 plan (*N* = 60) vs. calorie restriction	19–65	35–50	40.2 (9.2)	13.3	40.4 (3.8)

*Health Management Resources (HMR)*						
Anderson et al. [57]	RT of HMR (*N* = 22) vs. controls	20–65	30–39.9	50.5 (7.3)	22.7	35.8 (3.2)
Donnelly et al. [58]	RT of HMR with phone support (*N* = 25) vs. control			53	36	34.6
Donnelly et al. [58]	RT of HMR with clinic support (*N* = 27) vs. control			52	37	32.8
Furlow and Anderson [30]	Medically supervised HMR in consecutive patients (*N* = 117).		≥30	48.3 (11.9)	32	41.6 (9.7)
Furlow and Anderson [30]	Healthy Solutions HMR program in consecutive patients (*N* = 56).		≥30	47.9 (13.5)	37	38.0 (6.7)
Smith et al. [59]	RT of HMR with no weekly support (*N* = 28) vs. control	19–70	25–40	44.6 (10.4)	25	34.6 (3.8)
Smith et al. [59]	RT of HMR with limited weekly support (*N* = 28) vs. control	19–70	25–40	46.9 (12.1)	23	32.7 (4.4)

*Diet Chef*						
Mellor et al. [60]	RT of Diet Chef (*N* = 56) vs. self-directed diet (*N* = 58)	30–70	27–35	45.1 (9.7)	27	31.6 (2.4)

Abbreviations: HMR: Health Management Resources; JC: Jenny Craig; NHS: National Health Service; RC: Rosemary Conley; RT: randomized trial; SW: Slimming World; and WW: Weight Watchers. ^∗^Mean (standard deviation).

**Table 2:** Estimated standard deviation for LOCF and completers as a function of the mean weight loss for use in estimating the missing standard errors (SD/N^0.5^) for other commercial results.

	Intercept	Mean weight loss	Mean weight loss^2^
*LOCF*			
Weight loss (kg)	3.200	0.781	0.158
Weight loss (%)	4.226	0.824	0.132
BMI	1.038	0.761	0.428

*Completers*			
Weight loss (kg)	1.940	0.080	0.042
Weight loss (%)	3.481	0.389	0.049
BMI	0.561	−0.017	0.093

^2^ *P* ≤ 0.05.

**Table 3:** Additional weight loss (±SE) of JumpstartMD vs. published results for other commercial programs.

	LOCF			Completers		
	12-13 wk	24–26 wk	52 wk	12-13 wk	24–26 wk	52 wk
*Δweight (kg)*						
Calorie, food, meal plan^1^	−2.75 ± 0.02	−3.51 ± 0.11	−3.29 ± 0.17	−4.36 ± 0.03	−26.50 ± 0.11	−6.86 ± 0.09
Internet^2^	−3.94 ± 0.25	−4.53 ± 0.32	−7.60 ± 0.75	−5.45 ± 0.20	−7.53 ± 0.34	−8.30 ± 0.85
MBNP^3^	+0.76 ± 0.22	+0.37 ± 0.26	−0.67 ± 0.20	+0.46 ± 0.21	−1.72 ± 0.23	−2.85 ± 0.21
Prepackaged meals^4^				−3.56 ± 0.76	−4.10 ± 0.28	−3.24 ± 0.43
Meal replacement^5^	+0.06 ± 0.51	+3.59 ± 0.32	+5.7 ± 0.91	−1.29 ± 0.06	−0.91 ± 0.12	−0.76 ± 0.13

*Δ% weight*						
Calorie, food, meal plan^1^	−2.86 ± 0.03	−3.19 ± 0.11		−4.23 ± 0.04	−5.37 ± 0.13	−1.88 ± 0.21
Internet^2^	−3.95 ± 0.26	−4.56 ± 0.32	−7.89 ± 0.80	−5.65 ± 0.21	−7.79 ± 0.36	–10.06 ± 0.67
Prepackaged meals^4^			−2.30 ± 0.04	−1.66 ± 0.04	−1.74 ± 0.07	+0.11 ± 0.12
Meal replacement^5^	+1.22 ± 0.5	+3.78 ± 0.27		−1.19 ± 0.06	−1.26 ± 0.11	−11.44 ± 0.60

*ΔBMI (kg/m* ^*2*^)						
Calorie, food, meal plan^1^	−1.01 ± 0.01	−1.25 ± 0.04	−0.82 ± 0.20	−1.60 ± 0.02	−2.23 ± 0.05	−2.98 ± 0.15
Internet^2^	−1.41 ± 0.08	−1.65 ± 0.10		−1.89 ± 0.07	−2.43 ± 0.13	
Prepackaged meals^4^					−2.22 ± 0.42	−2.21 ± 0.70
Meal replacement^5^		−1.02 ± 0.36	−2.25 ± 0.32	−0.68 ± 0.16	−2.90 ± 0.39	−4.54 ± 0.40

*≥5% weight loss (%)*						
Calorie, food, meal plan^1^	26.02 ± 0.25	12.95 ± 0.74	17.14 ± 2.65	35.36 ± 0.39	15.74 ± 0.70	33.73 ± 1.27
Internet^2^	37.90 ± 2.67	32.1 ± 2.80		44.12 ± 2.46	46.86 ± 3.34	46.97 ± 7.00
MBNP^3^						17.09 ± 2.23
Prepackaged meals^4^						24.21 ± 3.57
Meal replacement^5^		−5.30 ± 2.65		−8.33 ± 0.50	−5.85 ± 0.55	1.68 ± 1.05

*≥10% weight loss (%)*						
Calorie, food, meal plan^1^	17.76 ± 0.25	22.9 ± 0.95		36.79 ± 0.50	30.65 ± 0.85	34.77 ± 1.47
Internet^2^				15.51 ± 3.51		
MBNP^3^						21.08 ± 2.33
Prepackaged meals^4^						25.84 ± 4.49
Meal replacement^5^		−1.92 ± 2.85		9.43 ± 0.47	6.52 ± 0.93	1.68 ± 1.29

Negative values means greater JumpstartMD weight loss for ∆weight, ∆%weight, and ∆BMI, positive values means greater JumpstartMD weight loss ≥5% and ≥10% weight loss. Individual study results are presented in Figures 5–15. ^1^Weight Watchers [6, 8, 11–14, 31–42], Slimming World [12, 13, 15–17, 20–22, 37, 43], Rosemary Conley [12, 13, 37, 42], and Itrim [26]. ^2^Biggest Loser Club [25, 44, 45] and Ediet [46, 47]. ^3^Metabolic Balance Nutrition Program (similar to JumpstartMD) [27]. ^4^Jenny Craig [23, 24, 3 and 3, 48–50] Diet Chef [60]. ^5^Nutrisystem [9, 33, 51–53], Medifast [28, 29, 54–56], Itrim [26], and Health Management Resources [30, 57–59].

## Data Availability

The data are not publicly available. Dr Bourke will consider reasonable requests for access to the data.
